# Oncolytic adenoviruses encoding bispecific T cell engagers or a novel trispecific T cell engager for dual-targeting of c-MET and EGFR

**DOI:** 10.1016/j.omton.2025.201106

**Published:** 2025-12-09

**Authors:** Martin A. Boos, Oliver Seifert, Stefanie Sawall, Jessica Genz, Annika Huber, Ilse Hofmann, Roland E. Kontermann, Guy Ungerechts, Dirk M. Nettelbeck

**Affiliations:** 1 Clinical Cooperation Unit Virotherapy, German Cancer Research Center (DKFZ), 69120 Heidelberg, Germany; 2 Institute of Cell Biology and Immunology, University of Stuttgart, 70569 Stuttgart, Germany; 3 Stuttgart Research Center Systems Biology, University of Stuttgart, 70569 Stuttgart, Germany; 4 Core Facility Antibodies, German Cancer Research Center (DKFZ), 69120 Heidelberg, Germany; 5 Department of Medical Oncology, Heidelberg University Hospital, Medical Faculty Heidelberg, Heidelberg University, and National Center for Tumor Diseases (NCT) Heidelberg, 69120 Heidelberg, Germany

**Keywords:** MT: Regular Issue, oncolytic adenovirus, virotherapy, immune evasion, dual-targeting, c-MET, EGFR, BiTE, single-chain diabody, trispecific T cell engager, solid tumors

## Abstract

This study reports a strategy to overcome immune escape of tumors during viro-immunotherapy due to tumor heterogeneity. We pursued a dual-targeting approach of epidermal growth factor receptor (EGFR) and cellular mesenchymal epithelial transition factor (c-MET) facilitated by two bispecific T cell engagers (TCEs) or one trispecific TCE encoded by oncolytic adenoviruses (oAds). Different bi- and trispecific TCE formats were generated and characterized. We showed efficacy of bispecific TCEs single-chain diabody (scDb)-cMET and tandem scFv (taFv)-EFGR *in vitro*, encouraging the design of the diabody-based trispecific TCE (Db-TriTE) scDb-cMET-scFv-EGFR. The Db-TriTE exhibited effective dual-targeting, i.e., the induction of cytotoxicity for single-positive cells of both targets and superior efficacy on double-positive cells. Therefore, a panel of oAds was produced to test different TCE (co-)expression strategies. Our results showed no or minor attenuation of viruses harboring the transgenes for one of the analyzed insertion strategies, expression of TCEs, and specific induction of both T cell activation and cytotoxicity in co-cultures of infected tumor cells with peripheral blood mononuclear cells (PBMCs). Importantly, we demonstrated dual-targeting by oAds encoding the two bispecific TCEs or the Db-TriTE with the latter featuring superior genomic stability and the overall highest efficacy. Taken together, we report novel bi- and trispecific TCEs and proof of principle for their application as dual-targeted viro-antibody therapy.

## Graphical abstract

**Figure:**
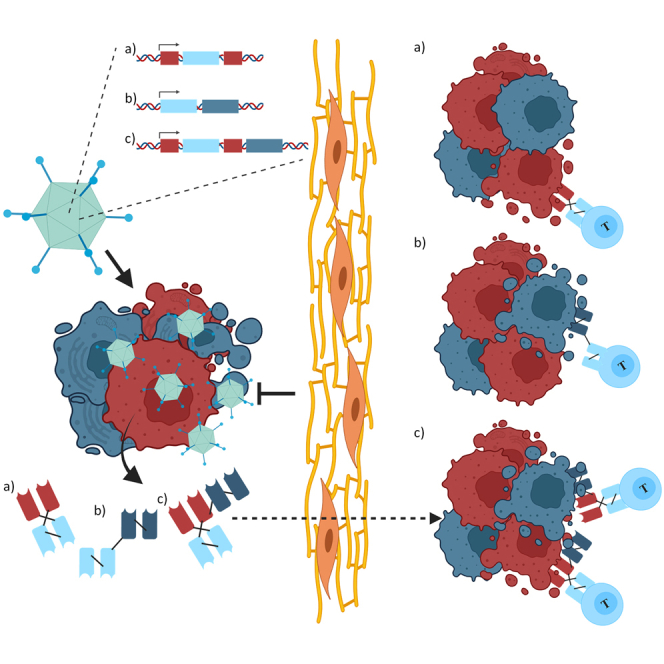

## Teaser

Targeting two distinct antigens via bi- or trispecific T cell engagers encoded by oncolytic adenoviruses is explored to address the problem of tumor heterogeneity. Different antibody formats and expression strategies are investigated *in vitro*, establishing a promising platform for next-generation, dual-targeted viro-immunotherapy.

## Introduction

Immunotherapy harnesses or complements the patients’ own immune system to kill cancer cells and has become a pillar of cancer therapy in clinical oncology in recent decades. Modalities include tumor-targeted monoclonal antibodies; genetically engineered chimeric antigen receptor- (CAR-) or T cell receptor- (TCR-) T cells for adoptive transfer^1^^,^^2^^,^^3^; immune checkpoint inhibitors, such as PD-1- and PD-L1-directed antibodies; and T cell engagers (TCEs) to recruit and activate the patients’ T cells and facilitate anti-tumor T cell action.^4^^,^^5^ Furthermore, oncolytic viruses are capable of triggering anti-tumor immunity through *in situ* vaccination resulting from targeted and immunogenic tumor cell (onco-) lysis.^6^^,^^7^ Increasingly, combination immunotherapies are being explored to increase efficacy and provide treatment benefit to more patients.^8^^,^^9^

Oncolytic viruses’ (OVs) fundamental mode of action is the selective infection and lysis of cancer cells, i.e., oncolysis.^6^^,^^10^ OVs have been derived from several human pathogenic viruses by engineering of viral genomes toward designer viruses to facilitate tumor-specific cell entry or post-entry replication.^10^^,^^11^^,^^12^ Alternatively, vaccine strains or viruses that do not naturally infect humans have been identified.^10^^,^^12^ These are sensitive to human cellular viral defenses and exploit defects in such host-defense pathways in cancer cells. Importantly, it is now established that viral oncolysis triggers anti-tumor T cell immune responses.^13^^,^^14^^,^^15^^,^^16^ These responses contribute critically to the therapeutic outcome and facilitate systemic therapy even after local OV application. Such immune responses are a result of an *in situ* vaccination effect, caused by the release of both danger signals and tumor (neo-)antigens from infected and lysed cancer cells. OVs have, therefore, emerged as promising cancer immunotherapeutics.

Another feature of several OVs that makes them especially attractive for (combination) immunotherapy of solid tumors is the opportunity to engineer their genomes to encode therapeutic proteins.^11^^,^^17^ Genetic delivery of effector proteins to tumors facilitates the rational modulation of the tumor’s immune microenvironment or the local delivery of immunotherapeutics,^11^^,^^12^^,^^18^ especially antibodies (viro-antibody therapy).^19^

T cell engagement uses bispecific adapter molecules, TCEs, to link T cells to cancer cells and thereby activate their effector functions independent of their intrinsic, TCR-mediated specificity.^5^ This forced interaction triggers T cell-mediated cancer cell destruction independent of effective intrinsic anti-tumor T cells. To this end, TCEs bind simultaneously to CD3 on T cells and to cancer cell surface molecules. In addition, TCEs are also being investigated for targeting tumor stromal cells, and related immune cell engagers redirect NK cells or macrophages to tumor cells.^20^^,^^21^^,^^22^^,^^23^ A small TCE molecule format links two single-chain variable fragments (scFvs) in tandem (tandem scFv [taFv]), for which the term BiTE (for bispecific TCE) has been established. An approved BiTE, blinatumomab, binds CD3 and the B cell lineage marker CD19 for treatment of B cell malignancies.^24^ However, BiTEs and related Fc-less molecules have limitations including rapid blood clearance due to their small size. Moreover, TCEs might have systemic side effects resulting from on-target-off-tumor cell binding.^25^^,^^26^^,^^27^ Furthermore, the treatment of solid tumors with TCEs faces significant challenges due to low tumor perfusion with TCEs and T cells and immunosuppressive tumor microenvironments.^25^

For several reasons, the delivery of TCEs by OVs (OV-TCE therapy) is an especially attractive match^28^: OV-encoded TCEs facilitate bystander killing of tumor cells not reached by the virus with small, Fc-less TCE formats facilitating better tumor penetration than larger IgG-like formats. The local and extended expression of Fc-less TCEs following genetic delivery by OVs addresses the abovementioned problems of systemically applied TCEs regarding pharmacokinetics and systemic side effects. Even more, TCEs redirect powerful OV-induced anti-viral T cell responses to cancer cells. Hence, the feasibility and efficacy of OV-TCE therapy for solid tumors has been extensively reported in preclinical models for OVs with taFvs (BiTEs) targeting tumor cells.^19^^,^^28^ Epidermal growth factor receptor (EGFR),^29^^,^^30^^,^^31^ EpCAM,^32^^,^^33^ MUC1,^34^^,^^35^ CD44v6,^36^^,^^37^ B7H3,^38^ EphA2,^39^^,^^40^^,^^41^ CEA,^42^ MUC16,^43^^,^^44^ B7H4,^45^ NKG2DL,^46^ Claudin 18.2,^47^ CD19,^48^ CD20,^49^ PD-L1,^50^^,^^51^ HER2,^52^ cancer-associated fibroblasts,^20^^,^^21^ or tumor-associated macrophages^53^ have been described as targets for OV-encoded taFvs. However, all reported TCE-encoding OVs have aimed at a singular tumor target.

Primary or secondary treatment resistance represents significant impediments to the efficacy of targeted drugs. Heterogeneity or plasticity of cancer cells with respect to targeted tumor surface markers has been demonstrated to reduce the efficacy of antibody-based cancer treatment modalities or to cause tumor relapse by antigen escape.^54^

Dual-targeting, the concurrent treatment with two targeted drugs, is a promising strategy to overcome treatment resistance through immunoediting.^55^^,^^56^^,^^57^ For solid tumors, dual-targeting of EGFR and cellular mesenchymal epithelial transition factor (c-MET) with a bispecific antibody, amivantamab, is approved for treatment of patients with non-small cell lung cancer (NSCLC) with an exon 20 insertion in the EGFR gene.^58^^,^^59^ However, it is associated with notable limitations regarding its safety profile stemming from the need of continuous systemic administration.^60^ The rationale for dual-targeting of both EGFR and c-MET besides heterogeneous expression is that an interplay between EGFR and c-MET expression is seen in cancer cells, where therapeutic targeting of one receptor leads to compensatory mechanisms and upregulation of the other receptor.^61^^,^^62^^,^^63^ While dual-targeting of EGFR and c-MET with small molecules has shown encouraging results, it shows a wide range of side effects that affect various organ systems.^64^

**Figure 1:**
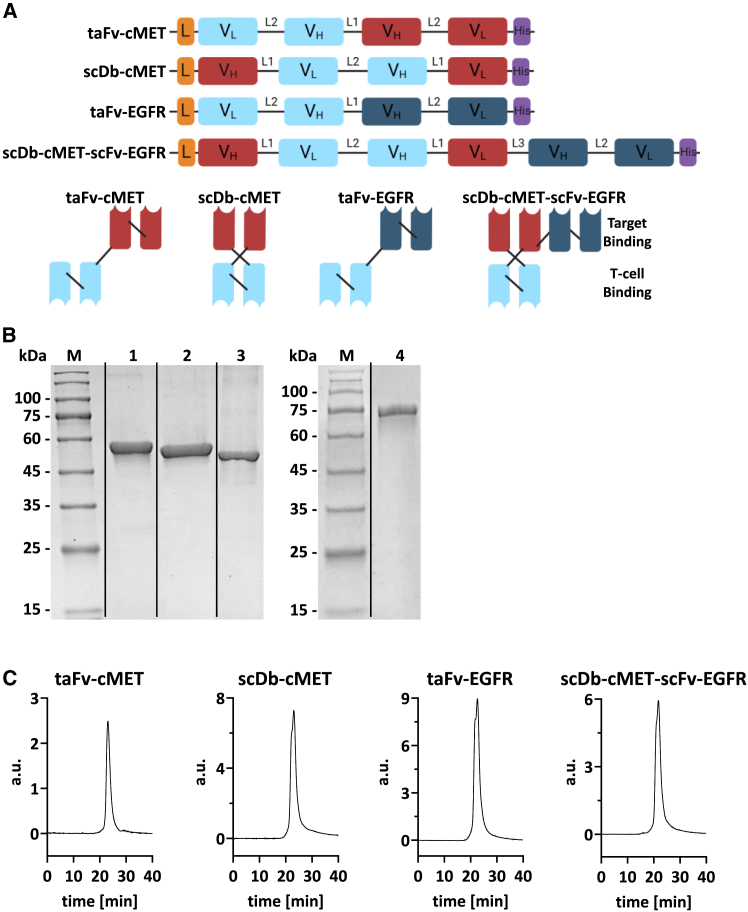
Biochemical characterization of bispecific scDb, taFv, and trispecific scDb-scFv antibody formats (A) Schematic illustration of taFv-cMET, scDb-cMET, taFv-EGFR, and scDb-cMET-scFv-EGFR at DNA (top) and protein levels (bottom). L, Igκ leader; V_H_, heavy chain; V_L_, light chain; L1, GGGGS; L2, (GGGGS)_3_; L3, AAAGGSGGGGS; His, 6x histidine tag. Light blue, huU3; red, c-MET; dark blue, EGFR. (B) SDS-PAGE analysis of antibody constructs. Lane 1, taFv-cMET; lane 2, scDb-cMET; lane 3, taFv-EGFR; lane 4, scDb-cMET-scFv-EGFR. Rearrangement of lanes indicated by black lines. 12% PAA, 1 μg/lane, Coomassie blue staining under reducing conditions. (C) Size-exclusion chromatography by HPLC using Superdex 200 Increase column. a.u, arbitrary unit.

The objective of this study was to develop and prove the principle of dual-targeted OV-TCE therapy. We explored different scFv-derived BiTE formats—notably taFvs and single-chain diabodies (scDbs)^65^—and a fusion of these formats to a novel dual-targeting trispecific scDb-scFv we termed *scDb-based trispecific TCE* (Db-TriTE). The rationale for comparing the TCE activity of scDbs to the established taFv (BiTE) format was our previously reported observation of significantly increased serum stability of the scDb format.^66^ This increase in stability most likely results from the folding of the scDb into a more compact and rigid molecule (Figure 1A), thereby reducing accessibility for proteolytic degradation (e.g., proteolytic separation of the two antigen-binding domains) and/or thermal degeneration. Moreover, a functional scDb TCE would allow for deriving the Db-TriTE that uses an scDb as building block. Consequently, for dual-targeting OV-TCE therapy, OVs need to be developed that either co-express two BiTEs or express a single Db-TriTE. Db-TriTEs possess advantageous features for OV-TCE dual-targeting, namely, the requirement of less genomic space in the OV genome and no risk of genomic recombination between two identical or similar anti-CD3 scFvs-coding sequences. For proof of principle, we focused on EGFR and c-MET as validated targets for dual-targeting therapy. We chose to produce OV-TCEs based on oncolytic adenoviruses (oAds), which are extensively investigated OVs in pre-clinical and clinical studies.^67^^,^^68^^,^^69^^,^^70^^,^^71^ Various advantageous properties recommend adenoviruses for applications as OVs, including lytic potency, particle stability, established manufacturing to high titers, knowledge of their genome organization and replication cycle facilitating virus engineering, and the capacity for the genomic insertion of larger heterologous DNA sequences.^71^^,^^72^^,^^73^^,^^74^ We and others have established effective strategies for the expression of one or more transgenes at various insertion sites in the viral genome and by different expression modalities.^74^^,^^75^^,^^76^^,^^77^^,^^78^ The present study aimed to identify an expression strategy that exhibited little to no attenuation of oAd-mediated oncolysis, retains genomic stability, and facilitates the expression of functional TCEs in therapeutically effective amounts. To this end, oAds encoding either two BiTEs or the Db-TriTE were generated toward proof of principle for dual-targeting viro-antibody therapy.

## Results

### The single-chain diabody as an alternative bispecific T cell engager format for oncolytic virus delivery

**Figure 2:**
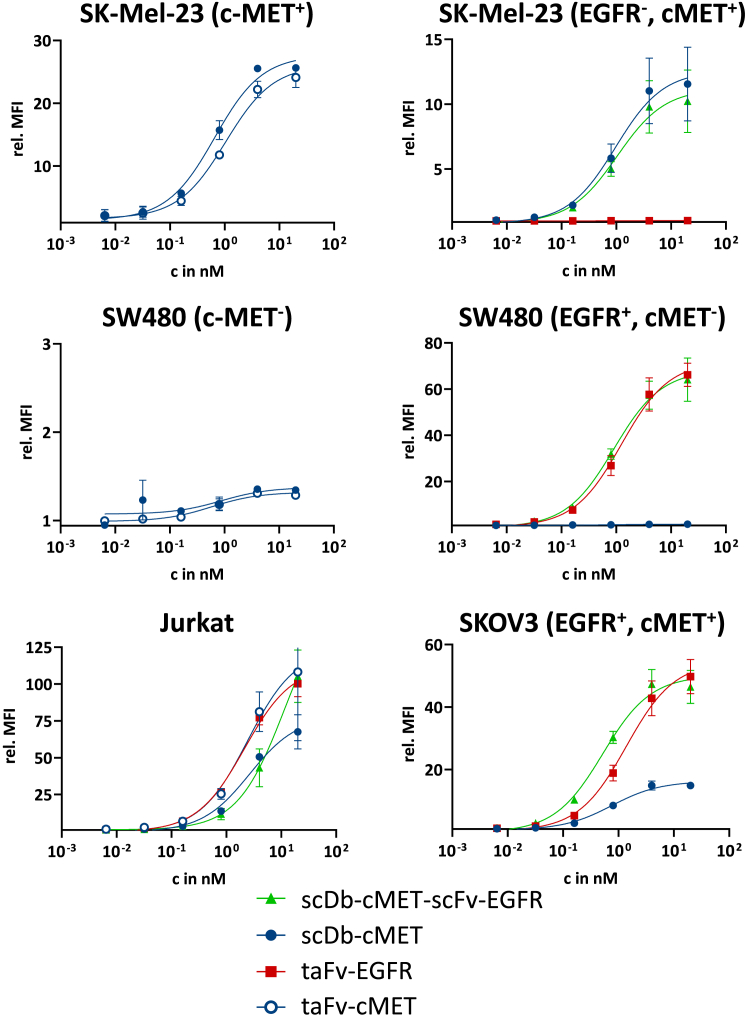
Binding properties of scDb-cMET, taFv-cMET, taFv-EGFR, and scDb-cMET-scFv-EGFR Comparison of cMET-binding strength and specificity of bispecific taFv and scDb formats in (A) SK-Mel-23 cells and SW480 cells. (B) Binding of scDb-cMET, taFv-cMET, taFv-EGFR, and scDb-cMET-scFv-EGFR to CD3^+^ Jurkat cells. (C) Binding of trispecific scDb-cMET-scFv-EGFR and parental bispecific scDb-cMET and taFv-EGFR to single- and double-positive target cells. Binding properties were analyzed via flow cytometry; bound protein was detected using PE-anti-His mAb. Relative MFI shown as mean ± SD, (*n* = 2).

**Figure 3:**
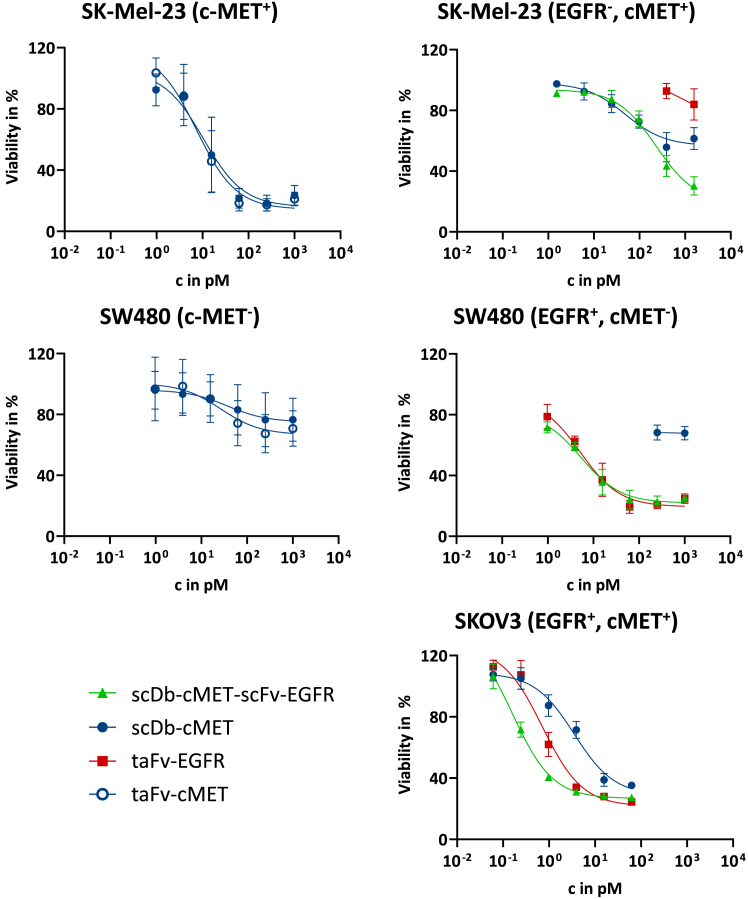
Induction of T cell-mediated cytotoxicity on single-positive and double-positive target cells Cells were incubated with a serial dilution of (A) scDb-cMET or taFv-cMET or (B) scDb-cMET, taFv-EGFR, or scDb-cMET-scFv-EGFR. PBMCs were added at an effector:target cell ratio of 10:1. Cell viability was measured 3 days after PBMC addition using crystal violet staining and subsequent quantification. Data shown as mean ± SD, (*n* = 3 donors, each determined in 3 independent experiments).

Previously, several OVs encoding single BiTEs have been reported, most of them encoding the clinically established taFv format (BiTE).^79^ We started our study with exploring scDbs as an alternative small TCE format, which differs in the order of V_H_ and V_L_ domains, resulting in a less-flexible and likely more stable format than the taFv (Figure 1A, we will use the term taFv rather than BiTE in the following, as the difference in molecular structure is relevant for our work). We thus started with the generation and comparative analysis of a taFv and an scDb for one of our favored targets, c-MET. To this end, we cloned expression plasmids in a previously reported design^65^ using V_H_ and V_L_ sequences of a humanized version of CD3 antibody UCHT1 and of a reported anti-c-MET Fab.^80^ Proteins were produced by transient transfection of FreeStyle-293F cells and purified by immobilized metal ion affinity chromatography (IMAC). SDS-PAGE analysis showed single bands of expected size (taFv-cMET: 56.56 kDa; scDb-cMET: 53.7 kDa), and the proteins eluted as single peaks in analytical size-exclusion chromatography not showing aggregates or fragments (Figures 1B and 1C). Comparative analysis of cell binding by flow cytometry with antibody titration revealed equal binding to c-MET^+^ target cells (SK-Mel-23; for antigen expression profiles of used cell lines, see Figure S1), equally weak binding to c-MET^−^ control cells (SW480) at high concentrations (Figure 2A), and somewhat stronger binding of the taFv-cMET to CD3^+^ T cells (Jurkat, Figure 2B). Finally, our comparative analysis of T cell-mediated tumor cell killing in co-cultures of tumor cells and peripheral blood mononuclear cells (PBMCs) demonstrated equal efficacy for taFv-cMET and scDb-cMET in co-cultures with c-MET^+^ target cells (Figure 3A). Thus, the difference in CD3 binding did not have an effect on induction of T cell-mediated cytotoxicity in co-cultures under the conditions we used. As expected from the binding analysis, comparable levels of weak induction of tumor cell cytotoxicity were observed in co-cultures with c-MET^−^ control cells at higher concentrations (Figure 3A). Taken together, we generated two novel TCEs targeting c-MET, and the comparison of the scDb and taFv formats showed equal efficacy of specific cell binding and T cell-mediated tumor cell killing for this target *in vitro*.

### A novel scDb-based trispecific T cell engager molecule for dual-targeting of c-MET and EGFR

For our aim of dual-targeting T cell engagement after genetic delivery by OVs, two approaches are feasible: the co-expression of two BiTE or the expression of one trispecific TCE. When using the same building blocks (e.g., CD3- and tumor-targeted scFvs), the latter approach has two important advantages. First, it needs less OV genomic capacity. Second, it reduces the risk of homologous recombination between identical or similar sequences that are required for encoding two CD3-binding moieties for the co-expression approach, but not for the trispecific format. Therefore, we hypothesized that a BiTE in the scDb format could be exploited as building block for a novel Db-TriTE by fusing a tumor-targeting scFv to the C terminus (Figure 1A). This follows our previously reported design of a trivalent but bispecific scDb-scFv.^81^ For c-MET/EGFR co-targeting, we fused the EGFR-targeted scFv to our scDb-cMET, generating the scDb-cMET-scFv-EGFR (Figure 1A). As this Db-TriTE basically constitutes an overlapping scDb and taFv, we used the corresponding bispecific scDb-cMET and taFv-EGFR as controls. The scDb-cMET-scFv-EGFR and taFv-EGFR proteins were produced and resulted in proteins of expected size (taFv-EGFR: 53.3 kDa; scDb-cMET-scFv-EGFR: 80.03 kDa, Figure 1B) without detectable fragments or aggregates (Figure 1C). Antigen binding was analyzed using both single and double target-positive tumor cell lines and Jurkat T cells. For CD3^+^ Jurkat T cells, we observed somewhat stronger binding with the taFv-EGFR than with the scDb-cMET (Figure 2B). The Db-TriTE showed binding similar to the scDb at lower concentrations but reached the median fluorescence intensity (MFI) of the taFv-EGFR at the highest concentration. Importantly, the Db-TriTE showed equal binding compared to the respective bispecific controls on single-positive target cell lines (Figure 2C), underlining its suitability for our dual-targeting strategy. Moreover, binding to double-positive cells was stronger for the Db-TriTE than for both bispecific controls (Figure 2C). Tumor cell killing assays in co-cultures with PBMCs showed that the Db-TriTE performed equally well to the bispecific control, taFv-EGFR on c-MET^-^/EGFR^+^ SW480 cells. On c-MET^+^/EGFR^−^ SK-Mel-23 cells, the induction of cytotoxicity by the Db-TriTE was equal for lower concentrations and visibly higher for higher concentrations (Figure 3B) compared to the scDb-cMET. Finally, on double-positive SKOV3 cells, induction of cytotoxicity by the Db-TriTE was significantly higher than for the bispecific controls, reflected by the EC_50_ values (Table 1). We conclude that the c-MET- and EGFR-targeted Db-TriTE performs equally to the parental BiTEs on single-positive target cells and shows superior binding and induction of T cell-mediated cytotoxicity on double-positive tumor cells, underlining its potential for genetic delivery by OVs and for dual-targeted T cell engagement.

**Table 1:** EC_50_ values of bispecific and trispecific TCEs for double-positive target cells

TCE	EC_50_ in pM	
scDb-cMET	3.54	
taFv-EGFR	0.72	
scDb-cMET-scFv-EGFR	0.16	

EC_50_ values for SKOV3 cells (data shown in Figure 3) were calculated using GraphPad Prism software by a dose-response nonlinear regression with a variable slope. EC_50_ values in pM as mean of 3 donors, each determined in three independent experiments, ∗∗*p* < 0.01, unpaired *t* test.

### Generation of oAds for genetic delivery of TCEs enabling dual-targeting of c-MET and EGFR

**Figure 4:**
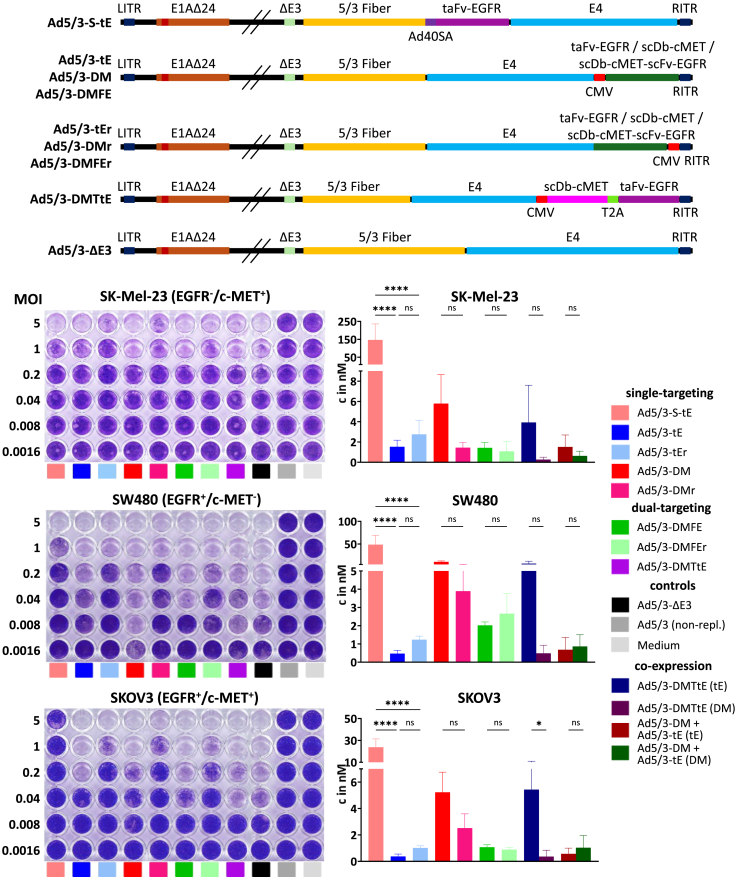
Oncolytic activity and antibody production of oAds (A) Schematic depiction of viral genomes. S, Ad40SA (splice-acceptor sequence of HAdV-40); tE, taFv-EGFR; DM, scDb-cMET; DMFE, scDb-cMET-scFv-EGFR; DMTtE, scDb-cMET-T2A-taFv-EGFR; r, reverse (right-to-left) orientation; LITR, left inverted terminal repeat; E1A, E3, and E4, early viral genes; Δ24, 24-bp deletion; ΔE3, deleted E3 region; 5/3 fiber, chimeric fiber with Ad5 tail and shaft and Ad3 knob; FSA, construct with splice-acceptor mediated expression downstream of fiber gene. (B) Cytotoxicity assays of oAds on SK-Mel-23, SW480, and SKOV3 cells. Target cells were infected with serial 5-fold dilutions of oAds (MOI = TCID_50_/cell). Cells were stained with crystal violet at time point of best visibility of differences between oAds. Representative experiments are shown. (C) Antibody concentrations in supernatants of infected target cells. Cells were infected with MOI 25 (SK-Mel-23) or MOI 5 (SW480, SKOV3) and supernatants were taken 7 days post infection and analyzed via flow cytometry with SKOV3 cells. Antibodies were detected using a PE-anti-His antibody or in case of dual-expression, AF647-anti-Myc or PE-anti-HA antibodies. Relative MFIs were used to calculate concentrations based on standard curves created by titration of purified antibodies. Values shown as mean ± SD, (*n* = 3), ∗*p* < 0.05, ∗∗∗∗*p* < 0.0001; ns, not significant; one-way ANOVA, for select comparisons.

Toward our aim of oAd-encoded T cell engagement targeting c-MET and EGFR, we next explored different approaches in order to find the most effective TCE expression and dual-targeting strategy. In principle, three strategies for dual-targeting are possible: co-infection (of two viruses encoding one BiTE each), co-expression (of two BiTEs by one virus), and the expression of a trispecific TCE. Moreover, for a given TCE, different insertion sites in the oAd genome and expression strategies are possible. Correspondingly, we generated a panel of TCE-encoding viruses for dual-targeting of c-MET and EGFR depicted in Figure 4A. As oAd backbone, we used Ad5/3Δ24ΔE3. This HAdV-5-derived virus encodes an E1A protein with deletion of the pRb-binding domain for tumor-restricted replication^82^ and has the E3 genes deleted to increase capacity for transgene insertion. Also, it possesses a fiber knob domain of HAdV-3 to enable efficient entry into tumor cells.^78^ As single expressor viruses, we inserted the cassettes encoding scDb-cMET, taFv-EGFR, or scDb-cMET-scFv-EGFR with a cytomegalovirus (CMV) promoter between the right inverted terminal repeat (RITR) and the E4 genes (“after-E4”^76^) in forward and reverse (“r”) orientation generating the 6 viruses Ad5/3-DM, Ad5/3-tE, Ad5/3-DMFE, Ad5/3-DMr, Ad5/3-tEr, and Ad5/3-DMFEr. For co-expression, we generated a virus with scDb-cMET and taFv-EGFR coding sequences linked with a T2A ribosomal skipping sequence and expressed from the CMV promoter in forward orientation, Ad5/3-DMTtE. The order of TCE sequences in this cassette was chosen as it resulted in best and similar expression of both TCEs in transient transfection experiments (not shown). With a final construct, Ad5/3-S-tE, we tested both an alternative insertion site in the oAd genome, downstream of the fiber gene (“after-fiber”), and an alternative expression strategy using a splice acceptor site for expression from the viral major late promoter, as reported before.^83^ All TCE-encoding viruses and the parental control virus without transgene, Ad5/3-ΔE3, could be rescued, amplified, and purified. However, the after-fiber virus Ad5/3-S-tE took longer time for rescue and amplification and yielded lower viral titers, which remained unchanged with repeated preparations and was also observed with insertions of other TCEs in this position (data not shown). Virus particle to TCID_50_ ratios were similar for all after-E4 viruses and the control (between 4 and 14, Figure S2) but was considerably higher for Ad5/3-S-tE (242, Figure S2).

### Oncolytic activity and TCE production by the panel of TCE-encoding oAds

We next investigated whether the insertion of TCE expression cassettes affected oncolytic activity of oAds, i.e., viral cytotoxicity in the absence of T cells, and how the insertion sites and expression strategies affect TCE expression by the oAds. To determine oncolytic activity, we infected our target tumor cell lines with serial dilutions of the 8 TCE-encoding viruses, the parental control virus Ad5/3-ΔE3, a non-replicating Ad5/3 virus, or mock (Figure 4B). The 4 after-E4 viruses with TCE expression cassettes in forward orientation, Ad5/3-DM, Ad5/3-tE, Ad5/3-DMFE, and Ad5/3-DMTBE, showed no attenuation (SK-Mel-23) or minor attenuation of less than 5-fold (SW480, SKOV3) compared to the control virus. Interestingly, the 3 after-E4 viruses with TCE expression cassettes in reverse orientation showed a further attenuation of approximately 5-fold, independent of the encoded TCE and cell line. This points to a general effect of the transgene insertion with a CMV promoter in reverse orientation at that site. Finally, the after-fiber virus showed the strongest attenuation in SW480 (25-fold) and SKOV3 (>100-fold) compared with the parental viruses. This was the case even though the assay was performed using infectious particles and thus already normalized for the lower infectivity of this virus.

To determine TCE expression, we infected our target tumor cells with our panel of TCE-encoding oAds and the control virus at multiplicity of infection (MOI) of 25 (SK-Mel-23) or 5 (SW480 and SKOV3). TCEs in supernatants were quantified 7 days post infection via flow cytometry, using standard curves generated with purified antibodies (Figure 4C). Notably, the after-fiber virus Ad5/3-S-tE showed highest TCE production in all cultures. taFv-EGFR production was significantly higher than for the two after-E4 taFv-EGFR-encoding viruses (Ad5/3-tE and Ad5/3-tEr). taFv-EGFR production was slightly higher for Ad5/3-tEr compared to Ad5/3-tE, although significance was not reached. In contrast, production of scDb-cMET was higher in oAds with the expression cassette in the forward orientation, also without reaching significance. For the Db-TriTE, insert orientation seemed to have no clear effect on the level of TCE production. For the after-E4 viruses, highest TCE concentrations were measured for oAds with scDb-cMET insertion (for SK-Mel-23 in forward orientation, only). In comparison, concentrations of taFv-EGFR and the Db-TriTE scDb-cMET-scFv-EGFR were lower and very similar in SK-Mel-23 and SKOV3 cells, whereas SW480 cells expressed scDb-cMET-scFv-EGFR somewhat stronger than taFv-EGFR.

We also determined TCE production by Ad5/3-DMTtE (co-expression via T2A) and after co-infection with Ad5/3-DM and Ad5/3-tE. For the latter, MOIs of 12.5 or 2.5 per virus were used to obtain the same total titer as for the other viruses. Note that scDb-cMET and taFv-EGFR TCEs in the supernatants could be distinguished via different tags. Unequal production of TCEs was observed for Ad5/3-DMTtE with dominance of taFv-EGFR (encoded downstream of the T2A sequence), which was even more strongly expressed than by the single-targeting Ad5/3-tE. In contrast, production of both TCEs was very similar in all tested cell lines after co-infection. Notably, an imbalance in T2A-mediated TCE co-production was not observed after plasmid transfection (data not shown). This observation and the fact that the transgene insert of Ad5/3-DMTtE included several homologous sequences prompted us to investigate potential recombination of the virus genome. Indeed, PCR analysis of purified Ad5/3-DMTtE revealed at least 3 deletions within the insert (Figure S3A). After further passaging of the virus in tumor cell cultures, especially the smaller PCR amplicons resulting from the larger deletions increased in intensity and a further even smaller amplicon and thus larger deletion mutant emerged. Sequencing of the PCR amplicons (Figure S3B) revealed recombination between identical or homologous sequences (1) encoding the leader peptides, (2) encoding the two CD3-specific V_H_ domains, (3) encoding the V_H_ domains of the c-MET- and EGFR-specific antibodies, and (4) of the CMV promoter and the V_L_ domain-encoding sequence of the downstream EGFR-specific antibody (least homology, but largest deletion). Hence, the deletions result in loss of the open reading frame of the upstream scDb-cMET (leader recombination) or of both antibodies (others). The former might contribute minimally to the observed imbalance of taFv versus scDb expression. These results encourage both the development of the Db-TriTE format (smaller insert and less-homologous sequences), as explored here, and the reduction of sequence homologies in the transgene insert of Ad5/3-DMTtE, e.g., by codon optimization and switching one of the leader peptides in future work. As the PCR analysis shows that the by far largest fraction of viruses in the Ad5/3-DMTtE preparation lacked detectable deletions and considering the mentioned opportunities for insert optimization, we included the virus in our further experiments.

To ensure that TCE expression during oAd replication does not compromise TCE bioactivity, we compared virus-produced with conventionally produced protein using the Db-TriTE as an example. Virus-encoded Db-TriTE was purified from the supernatant of Ad5/3-DMFEr-infected A549 cells. We observed no difference in binding to EGFR^+^/c-MET^+^ SKOV3 cells and only an insignificant reduction in inducing T cell-mediated cytotoxicity in co-cultures of SKOV3 cells and PBMCs (Figures S4A and S4B) implying no loss of bioactivity after oAd genetic delivery. High-performance liquid chromatography (HPLC) analysis revealed a similar peak for both proteins, however, with a small shoulder of high molecular weight species for the virally encoded Db-TriTE (Figure S4C). This might be due to differences in the first steps of TCE purification and needs to be further investigated for further development of this approach toward clinical translation.

In summary, TCE expression in the after-fiber position via splice-acceptor was very high compared to after-E4 expression from the CMV promoter, but came at the price of strongly attenuated oncolytic activity. Insert orientation in the after-E4 site seemed to have no clear effect on TCE expression, but insertion in the reverse orientation led to attenuation of oncolytic activity for all TCEs. Dual TCE expression by co-infection but not by co-expression via a T2A peptide was equimolar, and the latter strategy suffered from genomic instability that progressed with increasing viral replication cycles.

### TCE-encoding oAds specifically activate T cells in co-cultures and can implement dual-targeting

**Figure 5:**
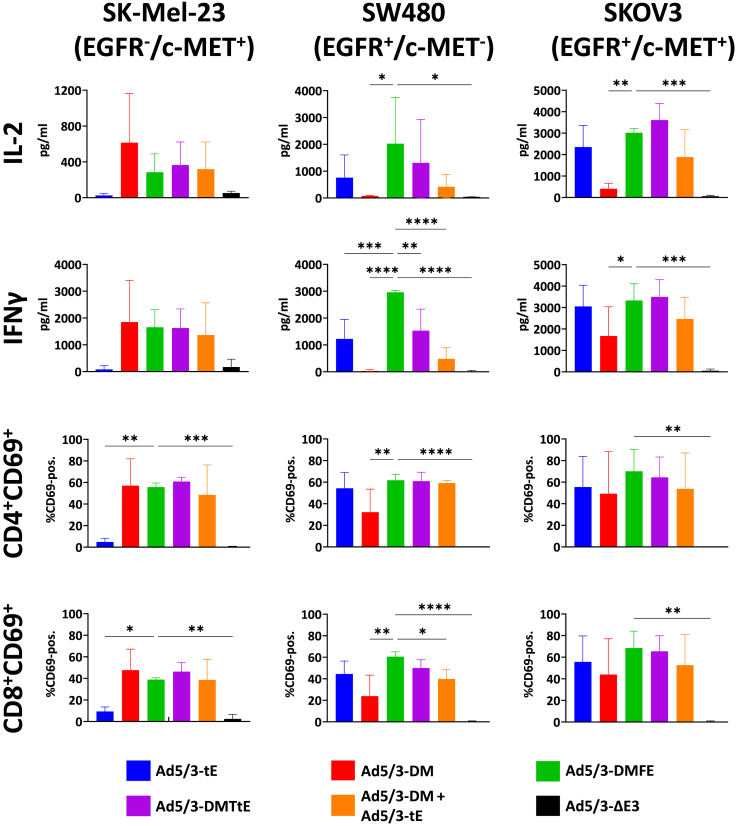
T cell activation by antibody-encoding oAds in co-cultures of infected tumor cells and PBMCs Tumor cells were infected with MOI 5 (SK-Mel-23) or MOI 1 (SW480, SKOV3). PBMCs were added 1 day post infection and IL-2 concentrations in the supernatant and CD69 surface expression were measured 24 h post addition of PBMCs. IFNγ concentrations in supernatants were measured 48 h post addition of PBMCs. Cytokine levels were measured via ELISA and CD69 expression via flow cytometry. Data gathered from 3 independent experiments with different donors, values shown as mean ± SD (*n* = 3), ∗*p* < 0.05, ∗∗*p* < 0.01, ∗∗∗*p* < 0.001, ∗∗∗∗*p* < 0.0001; one-way ANOVA. Only *p* values for significant differences of comparisons of Ad5/3-DMFE with other viruses are shown (complete analysis in Figure S5).

Having confirmed both production and bioactivity of virally encoded TCEs, we wanted to verify the potency of TCE-armed oAds for dual-targeting of EGFR and c-MET. As a first step, we analyzed T cell activation by TCE-armed oAds in co-cultures of oAd-infected tumor cells and PBMCs. Taking into consideration the TCE production and oncolytic activity data, we decided to focus our analysis on dual-targeting with the Db-TriTE-encoding virus (Ad5/3-DMFE), co-expression of scDb-cMET and taFv-EGFR (Ad5/3-DMTtE), and co-infection (Ad5/3-tE + Ad5/3-DM). Co-cultures infected with individual BiTE-encoding oAds (Ad5/3-tE, Ad5/3-DM) or the parental virus without TCE gene (Ad5/3-ΔE3) served as controls. We infected single target-positive cell lines SK-Mel-23 and SW480 and the double target-positive cell line SKOV3 with this panel of viruses. PBMCs of three different healthy donors were added 1 day post infection. Supernatants of co-cultures were taken for quantification of secreted cytokines interleukin (IL)-2 and interferon (IFN) γ. In addition, CD69 expression was measured on CD4^+^ and CD8^+^ T cells 24 h post addition of PBMCs (Figure 5 with statistics shown only for comparisons with Ad5/3-DMFE; for the full statistical analysis see Figure S5).

As expected, on single -positive EGFR^−^/c-MET^+^ SK-Mel-23 cells, Ad5/3-DM but not Ad5/3-tE triggered a significant induction of cytokine secretion and activation marker expression compared to Ad5/3-ΔE3 infection. Due to the relative insensitivity of this cell line to T cell killing, the effector-to-target (E:T) ratio was increased to 6:1 for SK-Mel-23, compared to 3:1 for the other cell lines. All dual-targeting oAds triggered the induction of cytokine secretion (not reaching significance) and activation marker expression (significant). There were no significant differences between dual-targeting strategies and Ad5/3-DM for any of the activation markers. On the other single-positive EGFR^+^/c-MET^-^ SW480 cell line, Ad5/3-tE triggered a strong increase in cytokine release and T cell activation marker expression compared to the control virus. Ad5/3-DM induced activation marker but not cytokine expression, which goes along with the strong TCE expression by this virus in these cells and the minor binding and T cell-mediated cytotoxicity observed for the scDb-cMET protein. Of note, all dual-targeting oAds induced increases in cytokine and activation marker expression with a trend toward weaker activity of the co-infection strategy and strongest activity of the Db-TriTE-encoding virus Ad5/3-DMFE. The latter significantly elevated cytokines and CD69 compared to the control virus and to Ad5/3-DM. Moreover, Ad5/3-DMFE was significantly superior to all other viruses regarding IFNγ secretion and to the co-infection strategy regarding CD69 expression on CD8^+^ T cells. The co-expression strategy with Ad5/3-DMTtE virus led to significantly higher IFNγ secretion and CD69 expression compared to Ad5/3-DM and Ad5/3-ΔE3. In addition, it led to a significant increase in IFNγ secretion compared to co-infection with Ad5/3-tE and Ad5/3-DM. Note that for interpretation of these results, antibody concentrations need to be considered (see Figure 4). For example, the Db-TriTE was stronger expressed after infection of SW480 cells with Ad5/3-DMFE than the taFv-EGFR after infection with Ad5/3-tE. Also, taFv-EGFR expression in all cell lines was stronger after infection with Ad5/3-DMTtE than after infection with Ad5/3-tE.

For double-positive SKOV3 cells, infection with all TCE-encoding oAds viruses led to a significant increase of CD69 surface expression of both CD4^+^ and CD8^+^ T cells compared to the control virus. In both cases, infection with Ad5/3-DMFE reached the highest level of significance. Secretion of cytokines IL-2 and IFNγ was also significantly elevated after infection with all viruses compared to the control virus, except for Ad5/3-DM for which the increase did not reach significance. This corresponds with the lower binding and T cell activation observed for the scDb-cMET protein for this cell line. There were no significant differences between dual-targeting strategies for any of the markers, except for significantly higher IL-2 secretion after Ad5/3-DMTtE infection compared to co-infection with Ad5/3-tE and Ad5/3-DM.

We conclude that only oAds implementing dual-targeting TCE strategies resulted in robust activation of T cells in co-cultures with both single-positive tumor cells and the Db-TriTE-encoding virus Ad5/3-DMFE showing the best results. Infection with the oAd encoding the non-cross-linking TCE led to none or little T cell activation. All TCE-encoding oAds triggered T cell activation in co-cultures with double-positive target cells, albeit with an overall lower T cell activation for the single-targeting Ad5/3-DM. Our results indicate efficacy of dual-targeting approaches using TCE-armed oAds including the Db-TriTE-encoding virus with respect to T cell activation.

### TCE-encoding oAds combine oncolysis and T cell-dependent killing in co-cultures of target cells and PBMCs

**Figure 6:**
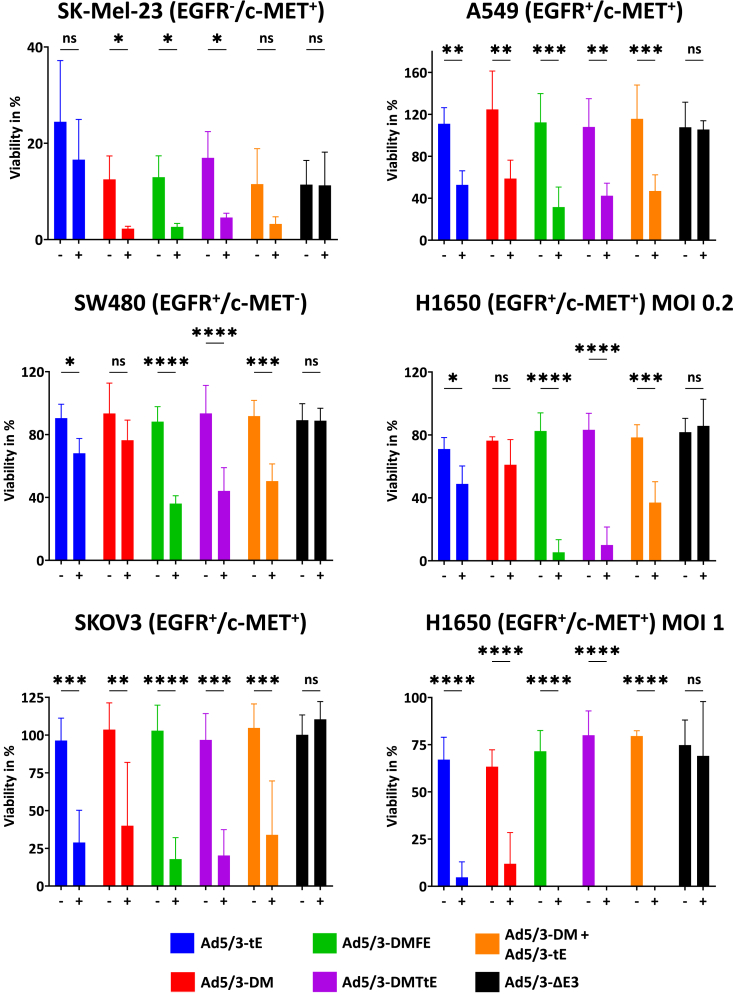
Combined oncolytic and antibody-mediated tumor cell cytotoxicity of antibody-encoding oAds in co-cultures of infected tumor cells and PBMCs (A) Cytotoxicity in single- and double-positive target cells. SK-Mel-23 cells were infected at an MOI of 25, SW480 cells at an MOI of 0.016, and SKOV3 cells at an MOI of 0.2. For SK-Mel-23 and SKOV3 cells, PBMCs were added 1 day post infection, and for SW480 cells, PBMCs were added 3 days post infection. Effector to target cell ratios were 10:1 for SK-Mel-23 and SW480 cells and 3:1 for SKOV3 cells. (B) Cytotoxicity in NSCLC cell lines. A549 cells were infected at an MOI of 0.04 and H1650 cells at MOIs of 0.2 and 1. PBMCs were added 1 day post infection with an effector to target cell ratio of 3:1. Cell viability was measured via crystal violet staining 6 days post infection. Data gathered from 3 independent experiments with different donors, values shown as mean ± SD (*n* = 3), ∗*p* < 0.05, ∗∗*p* < 0.01, ∗∗∗*p* < 0.001, ∗∗∗∗*p* < 0.0001; one-way ANOVA. “−”, without PBMCs; “+”, with PBMCs. Note the different scales of the *y* axes.

Last, we analyzed the combined effect of oncolysis and T cell-mediated cytotoxicity via TCE-dependent dual-targeting. Tumor cell killing was analyzed in co-cultures of oAd-infected single- or double-positive tumor cells in the presence or absence of PBMCs. This stringent assay requires an adequate potency of T cell-mediated tumor cell killing in the presence of viral oncolysis. As we observed differences in sensitivity to T cell killing between cell lines, we adjusted virus doses, E:T ratios, and time points of PBMC addition for SK-Mel-23 cells and SW480 cells. Results of the cytotoxicity analysis with three independent PBMC donors are shown in Figure 6A.

For SK-Mel-23 cells, infections were performed at high virus titers (MOI 25) and high E:T ratios (10:1) to address lower infectivity and especially lower sensitivity to T cell-mediated cell killing. In these EGFR^−^/c-MET^+^ cells, the viruses that did not drive production of a c-MET-targeting TCE did not lead to a significantly reduced viability in the presence of PBMCs. Also, co-infection with Ad5/3-tE and Ad5/3-DM failed to significantly increase cytotoxicity in the presence of PBMCs. Importantly, we observed combined oncolysis and T cell-mediated cell killing, i.e., a significantly increased cytotoxicity in presence of PBMCs, for Ad5/3-DM and for the dual-targeted viruses Ad5/3-DMFE and Ad5/3-DMTtE. For SW480 cells, the protocol was adjusted to an MOI of 0.016, an E:T ratio 10:1, and addition of PBMCs 3 days post infection. For these EGFR^+^/c-MET^−^ cells, no significantly enhanced cytotoxicity was observed in co-cultures where no EGFR-targeting TCE was produced. In contrast, a significant increase in cytotoxicity by addition of PBMCs was observed for viruses Ad5/3-tE, for co-infection with Ad5/3-tE and Ad5/3-DM, and for the dual-targeting viruses Ad5/3-DMFE and Ad5/3-DMTtE. The Db-TriTE-encoding virus Ad5/3-DMFE showed the strongest cytotoxicity. Together, these results demonstrate superiority of dual-TCE-targeting, as only the dual-targeting TCE-encoding oAds Ad5/3-DMFE and Ad5/3-DMTtE showed significantly increased cytotoxicity in the presence of PBMCs for both single-target tumor cell lines. The double-positive SKOV3 cells showed the highest sensitivity to TCE-mediated killing. Therefore, we detected a highly significant increase in cytotoxicity for all TCE-encoding oAds already at titers far below the titers required to show oncolytic cytotoxicity. Again, the strongest and most significant effect was observed for the Ad5/3-DMFE.

Finally, we investigated the potency of our set of TCE-encoding oAds for T cell-mediated killing of NSCLC cells, as dual-targeting of c-MET and EGFR is clinically established for the treatment of NSCLC patients, but modalities with reduced systemic side effects are warranted.^58^^,^^59^^,^^60^ To this end, we performed co-culture infection experiments with A549 and H1650 cells and PBMCs from 3 independent donors. Both cell lines express c-MET and EGFR (Figure S1). Our results revealed potent TCE-mediated killing for all antibody-encoding viruses in both cell lines (Figure 6B). In A549 cells, we detected a similar potency for this set of viruses with the Db-TriTE-encoding Ad5/3-DMFE showing a trend toward highest activity. This cytotoxicity profile matches the results for SKOV3 cells. In H1650 cells, all c-MET/EGFR-dual-targeting approaches, i.e., co-infection, co-expression, or expression of the trispecific Db-TriTE, were superior to mono-targeting of EGFR and c-MET. Mono-targeting, i.e., infections with oAds encoding either taFv-EGFR or scDb-cMET, required higher virus titers to achieve potent/significant T cell-mediated killing. These results confirm the potency of the dual-targeting TCE viro-antibody therapy approach in NSCLC cells.

In summary, our cytotoxicity data show increases in cell killing by oAd-encoded TCEs specifically for cell lines that are positive for the TCE target. More importantly, the dual-targeting effect observed for T cell activation (Figure 5) translates into TCE-mediated cell killing during oncolysis: killing of both cell lines expressing one TCE-target requires dual-targeting by co-infection or by the Db-TriTE or the co-expression strategy implemented with our novel oAds Ad5/3-DMFE and Ad5/3-DMTtE, respectively.

## Discussion

Dual-targeting strategies are increasingly investigated as a means to overcome treatment resistance and enhance the efficacy of cancer immunotherapies. By simultaneously targeting two different antigens, these approaches aim to improve treatment responses of heterogeneous cancers and prevent antigen escape. In this study, we focused on dual-targeting via antibodies, specifically TCEs, genetically delivered by oAds using the validated targets EGFR and c-MET for proof of principle.

We investigated two BiTE formats, namely, scDb and taFv. The only direct comparison of the two formats performed to date explored their application as bispecific adapters for entry targeting of adenoviral vectors.^66^ This study showed that binding properties of scDbs and taFvs were identical, the only difference being higher stability of the scDbs under physiological conditions,^66^ which would also be advantageous for applications as TCEs. Our results show equal efficacy as TCEs for scDb-cMET and taFv-cMET (Figures 2 and 3), while we previously found that the scDb-EGFR is ineffective, suggesting target epitope-specific limitations of the scDb format for applications as TCEs. This might be due to the lower flexibility of the scDb format that would not influence binding to either the tumor target or CD3 but could lead to steric hindrance when both antigen-binding sites of the scDb are to be engaged at the same time. Work by Asano et al. comparing different domain orders for diabodies has shown that binding affinities were not notably influenced by V_H_/V_L_ domain rearrangement, but significant differences in cross-linking efficacies were observed. This was not the case for taFv molecules, where antigen binding sites can rotate freely.^84^ In summary, we engineered novel c-MET-targeted TCEs in both the taFv and scDb formats and showed their potency *in vitro*, thereby facilitating future translation as therapeutics for (combination) treatment of c-MET-positive cancers.

Building on the encouraging data for scDb-cMET, we generated a novel dual-targeting Db-TriTE, using the scDb-cMET as a base and fusing it to the scFv-EGFR of the taFv-EGFR (Figure 1A). This TCE format was inspired by our previous work on a trivalent BiTE, targeting HER3,^81^ which exhibited increased binding to HER3^+^ cancer cells compared to the corresponding scDb, resulting in increased T cell activation, proliferation, and induction of T cell-mediated killing. Furthermore, a trivalent scDb-scFv molecule was reported linking a bispecific scDb, binding to PD-L1 and 4-1BB for T cell co-stimulation, to a human serum albumin-binding scFv for increased plasma half-life.^85^ Our novel Db-TriTE, scDb-cMET-scFv-EGFR, demonstrated equal binding and induction of cytotoxicity to its bispecific controls on single-positive target cells and superior activity on double-positive target cells. While the primary purpose of the Db-TriTE TCE format (and of our dual-targeting approach in general) is to ensure killing of both types of single-positive target cells in order to avoid antigen escape, its superior efficacy on double-positive target cells may be of further advantage. Potential mechanisms underlying the latter include an increased avidity of tumor cell binding resulting in a stronger and/or more persistent cell linkage. This is supported by the somewhat stronger cell binding compared to the better of the bispecific constructs, the taFv-EGFR (Figure 2C). For tumor cell populations that feature heterogeneous expression of the individual target antigens, the Db-TriTE might bind to tumor cells more homogeneously. Moreover, in comparison to two BiTEs applied in combination (not explored here), the “high-dose hook effect” might apply (see below and Roy et al.^98^). Overall, our results for the recombinant Db-TriTE, scDb-cMET-scFv-EGFR demonstrate the beneficial effects of dual-targeted T cell engagement. Our results are in line with encouraging findings for other small (Fc-less) trispecific single-chain TCEs targeting two cancer antigens of solid tumors. These include a nanobody-based trispecific TCE consisting of three fused nanobodies; heavy-chain-only antibodies (V_HH_), targeting CD3, FAP, and PD-1^86^; or a trispecific antibody named DTriTE, constructed via fusion of 3 scFvs specific for CD3, IL13Rα2, and EGFRvIII.^87^ Thus, our study stimulates the engineering of Db-TriTEs targeting further antigen combinations and the comparative analysis with other trispecific TCE formats, especially the DTriTE.

Numerous studies have successfully implemented TCE delivery to tumor cells via OVs,^19^^,^^28^ but an OV driving expression of multiple TCEs or a trispecific TCE for dual-targeting has not been described to date. Here, we report a panel of oAds for dual-targeting of EGFR and c-MET, investigating different TCE expression strategies and dual-targeting approaches. First, we explored different transgene insertion sites within the oAd genome, with the rationale to later insert two TCE genes in different insertion sites. We observed the highest TCE expression for transgene insertion in the after-fiber site, as determined for Ad5/3-S-tE encoding the taFv-EGFR, in comparison to all oAds encoding the taFv-EGFR in the after-E4 site. However, this virus was produced with low infectivity and showed a considerable attenuation of oncolytic potency. A previous study directly compared expression from the after-fiber site with the after-E4 site with the same expression strategy and found that the after-fiber site showed enhanced transgene expression that came at the expense of oncolytic potency.^88^ This would be in line with our findings here, although other studies, including our own previous work, did not show this attenuation for adenoviruses expressing reporter genes or a prodrug convertase via an internal ribosome entry site (IRES) or a splice-acceptor sequence in the after-fiber position, suggesting a dependency of the attenuation on the insert sequence, e.g., on its GC content or codon usage.^76^^,^^78^^,^^88^^,^^89^^,^^90^^,^^91^ All other oAds reported here expressed TCEs via a CMV promoter in the after-E4 site, where we revealed a link between insert orientation and oncolytic activity but no clear association with transgene expression (Figures 4B and 4C). Our observation of reduced oncolytic potency of oAds with CMV-driven transgenes in the after-E4 site, specifically in the reverse orientation, is to our knowledge the first report of such an orientation effect in that site. Previous studies by others and us have explored transgenes expressed from a splice acceptor site in this position,^76^^,^^92^^,^^93^ which required insertion in the forward orientation to enable expression from the major late promoter. Oncolytic potency was reported to be reduced approximately 5-fold^76^ or unchanged.^93^ Our result of orientation-dependent attenuation of oncolytic potency should be considered for future engineering of “armed” oAds. While the underlying mechanism remains to be revealed, possible explanations include (1) attenuating effects of readthrough transcription from inserted heterologous promoters (transcription competition or antisense effects), (2) inhibition of required readthrough transcription from viral elements by the inserted polyA signal sequence, (3) interference of the inserted heterologous promoter with viral promoters (might depend on proximity of elements and thus insert orientation), and (4) interference of cryptic splice sites with virus splicing in case of readthrough transcripts. Certainly, transgene inserts might also have orientation-independent effects, such as the destruction or separation of viral elements or the competition of strong transgene expression with resources needed for viral gene expression, such as transcription factors or tRNAs.^74^ However, in our study the orientation of the transgene cassette did not in all cases affect expression strength and oncolytic potency in opposing direction. Overall, our results go along with the general observation (independent of the insertion position) that the level of viral replication of armed oAds depends on the transgene, the promoter, and the cassette orientation.^74^

For the oAd co-expressing the scDb-cMET and the taFv-EGFR via T2A-peptide (Ad5/3-DMTtE), an imbalance in TCE expression was detected. This imbalance was not seen following transient transfection of cells with the expression plasmid (data not shown). Unexpectedly, we observed stronger expression of the taFv encoded downstream of the 2A peptide, while previous studies report stronger expression of the upstream sequence.^94^^,^^95^ In consequence, this strategy needs further optimization with respect to enabling similar expression levels, e.g., by testing different 2A sequences. Alternative co-expression strategies include insertion into different loci, the use of IRESs, or the use of bidirectional promoters.^88^^,^^96^^,^^97^ Our results for insertion of the TCE expression cassette in the after-fiber site showing clear reduction of oncolytic activity led us to prioritize the after-E4 site for exploring co-expression strategies and disfavored co-expression by using both the after-fiber and after-E4 sites. The IRES strategy was not pursued here, due to the considerable size of the element compared with 2A sequences. However, we explored bidirectional promoter expression cassettes but observed reduced oncolytic activity, genome instability, and unequimolar TCE expression for these oAds (data not shown). For our Ad5/3-DMTtE co-expressing scDb-cMET and taFv-EGFR via the T2A-peptide, we observed genomic instability. Specifically, we detected various deletions by homologous recombination in the antibody-encoding insert. While one of the deletions results in loss of the scDb but not the taFv coding sequence, its low prevalence in the used virus preparation cannot explain the above-discussed imbalance in scDb versus taFv expression. However, the detected genomic instability of Ad5/3-DMTtE has important implications for the future development of therapeutic protein-encoding, especially antibody-encoding oAds. First, our observation confirms that a strong increase of the adenoviral genome size by transgene insertion (Ad5/3-DMTtE: 104% of wild-type genome) should be avoided as it imposes a selection pressure to delete insert sequences. In this respect, our oAd encoding the trispecific Db-TriTE (Ad5/3-DMFE) is an advantageous dual-targeting format (101% of wild-type genome). Second, repeats of elements of identical sequences or strong homology should be avoided in (multi-) transgene inserts. Ad5/3-DMFE also addresses this problem as it lacks second copies of sequences coding for the leader peptide and the CD3-specific V_H_ and V_L_ domains that are present in the co-expressing Ad5/3-DMTtE. Thus, we propose that future research toward inserting multiple transgenes (especially antibodies) into oAd genomes should avoid homologous sequences (1) by rather combining sequence-different elements (e.g., promoters, polyA signals, or leader sequences) and/or (2) by codon optimization toward reduced sequence homology (e.g., for sequences encoding the framework regions of humanized antibody domains, even when expressing single TCEs like BiTEs). By such means, also the dual-targeting viruses reported in our study (Ad5/3-DMTtE and Ad5/3-DMFE) can be further optimized in future work. In conclusion, our study shows proof of principle for oAds co-expressing two TCEs via a 2A sequence; however, this approach would benefit from further optimization. The mentioned limitations of this co-expression strategy support the use of the Db-TriTE format for dual-targeting TCE-encoding oAds.

Compared to a combination of two BiTEs, the Db-TriTE is not only of advantage in consideration of genomic stability but also less likely to become subject to the high-dose hook effect,^98^ i.e., at high concentrations within the tumor that could be reached during phases of TCE-armed oAd replication, binding sites on both target cells and T cells could become saturated, reducing the formation of immune synapses. This effect would be observed at lower concentrations of two BiTEs compared to one trispecific TCE, due to the additional CD3-binding domain. We demonstrate expression of the oAd-encoded Db-TriTE scDb-cMET-scFv-EGFR after insertion of the transgene into the after-E4 site. The virus-encoded Db-TriTE did not show significant differences in target binding and induction of cytotoxicity compared to the protein produced by transfected cells (Figures S4A and S4B).

Finally, we analyzed T cell activation and cytotoxicity of the generated TCE-encoding oAds in co-cultures with PBMCs. As we observed differences in sensitivity to T cell-mediated cytotoxicity between our target tumor cell lines, we adjusted conditions of the cytotoxicity assays for each cell line so that the T cell-mediated effect was not masked by strong oncolytic effects. Similar setups for characterization of single-targeting BiTE- (i.e., taFv-) armed oAds have been previously described.^29^^,^^30^^,^^31^^,^^32^^,^^34^^,^^35^ We observed specific T cell activation, determined by cytokine secretion and expression of activation markers in both CD4^+^ and CD8^+^ T cells, and induction of T cell-mediated cytotoxicity in co-cultures when TCEs with specificity for targets expressed on the tumor cells were produced. No clear correlation between strength of TCE expression and T cell activation was observed, but there was a trend toward higher T cell activation with stronger TCE production and higher expression of the corresponding target antigen on co-cultured tumor cells. Importantly, T cell activation and cytotoxicity data showed that a dual-antigen-targeting approach using TCE-armed oAds was feasible. All tested dual-targeting strategies activated T cells and induced T cell killing in addition to viral oncolysis on single target- and double target-positive cell lines. For single target-positive cell lines, no significant loss in potency was observed when comparing dual-targeting versus single-targeting TCE-encoding oAds (or even superior activity due to stronger TCE expression as for Ad5/3-DMFE in SW480 cells). Across all tested cell lines, the Db-TriTE-encoding Ad5/3-DMFE exhibited the highest overall potency. This was encouraging considering that the Ad5/3-DMFE offered further notable advantages compared to either co-infection or co-expression approaches, i.e., stable equimolar targeting of both antigens and genomic stability. While OV-BiTE therapy has entered clinical investigation with an oAd encoding an FAP-specific BiTE and further immunostimulatory proteins (PsiOxus, virus NG-641, NCT 04053283), the problem of immune escape has not been addressed, so far. This phenomenon has been observed, e.g., for CD19 CAR-T cell therapy in patients with hematopoietic cancers and is likely to be a more significant challenge in solid tumors due to their pronounced heterogeneity.^99^^,^^100^ Our results recommend OV-encoded dual-targeting antibodies and more specifically the Db-TriTE-encoding oAd Ad5/3-DMFE for dual-targeting of EGFR and c-MET frequently co-expressed in NSCLC, glioblastoma, and colorectal cancer.

The increased potency or durability of treatment responses of dual-targeting strategies has been demonstrated for antibody-based interventions, such as therapeutic antibodies,^101^ BiTE therapy,^102^^,^^103^ and CAR-T cell therapy.^104^^,^^105^^,^^106^^,^^107^^,^^108^^,^^109^ Moreover, a dual-targeting approach combining BiTE- and CAR-mediated T cell therapy has been reported,^110^ as have the synthetic DNA delivery of two TCEs.^111^ Currently, bispecific CAR-T cells are being studied in clinical trials for the treatment of hematopoietic and solid cancers.^112^^,^^113^ In addition to the combination of two TCEs, the potency of dual-targeting by trispecific TCEs and NK cell engagers, which are based on IgG, scFv, and nanobody formats, has been demonstrated in preclinical models of hematopoietic and solid cancers.^87^^,^^114^^,^^115^^,^^116^^,^^117^^,^^118^^,^^119^^,^^120^^,^^121^^,^^122^^,^^123^^,^^124^^,^^125^ Our present study extends the dual-targeting strategy to OV-encoded TCEs.

Based on the proof of principle provided here for oAd-mediated dual-targeting TCEs, future work will have to explore the activity of the scDb-cMET- and taFv-EGFR-encoding Ad5/3-DMTtE and of the Db-TriTE scDb-cMET-scFv-EGFR-encoding Ad5/3-DMFE in heterogeneous primary tumor cultures and animal tumor models (for which, however, the Ad5/3-DMTtE should first be improved for better co-expression as discussed above). Of note, previous studies have shown therapeutic potency of oAds encoding TCEs targeting single tumor antigens in several tumor models,^20^^,^^29^^,^^30^^,^^31^^,^^33^^,^^34^^,^^35^^,^^36^^,^^37^^,^^38^^,^^39^^,^^40^^,^^41^^,^^42^^,^^43^^,^^44^^,^^47^^,^^48^^,^^49^^,^^51^^,^^52^ demonstrating that the approach, in general, succeeds in overcoming the potential barriers of the tumor microenvironment to T cell infiltration and therapeutic effectivity. Future studies based on the results reported here can investigate the safety profile of the OV-mediated EGFR/c-MET dual-targeting approach. This work should especially consider systemic toxicities that were reported for amivantamab in patients, mainly dermatological adverse events, including acneiform eruptions, widespread inflammatory papules, and dermatitis.^60^ Many other therapeutics that are administered systemically face similar challenges due to their side effects in healthy tissues, as described, e.g., for ICIs.^126^ Local production of antibodies via OVs can greatly reduce the systemic side effects that arise following intravenous application, overcoming a crucial challenge in immunotherapeutic approaches.^127^

Furthermore, our approach facilitates dual-targeting by TCE-encoding oAds to further target marker combinations, including those co-targeted by other therapeutic approaches, e.g., EGFR and HER2,^128^^,^^129^ HER2 and IL13Rα2,^105^ or EGFR and IL13Rα2.^110^^,^^112^ Such approaches might require optimization of the TCE format and of oAd-mediated (co-)expression, as discussed above.

In conclusion, our study further encourages the use of TCE-armed oAds for cancer immunotherapy demonstrating that dual-TCE-targeting via oAds is feasible and effective *in vitro*. To this end, we introduce a novel trispecific TCE antibody format, Db-TriTE. A first representative binding to both EGFR and c-MET shows dual-targeting potency as purified protein and when encoded by an oAd. Our approach suggests further applications of Db-TriTE-encoding oAds for targeting of different antigen combinations and cancer entities.

## Material and methods

### Antibody cloning, production, and purification

scDb-cMET and taFv-cMET were generated using variable domains of the 5D5 antibody^80^ and a humanized version of UCHT1 (huU3). For the taFv-EGFR, humanized versions of the variable domains of cetuximab (hu225) were used. The variable domain-coding sequences were cloned into a modified pSecTagA vector (Invitrogen, Thermo Fisher Scientific, Waltham, MA, USA, V90020) in the order and with linkers as depicted in Figure 1A. The scDb-scFv was generated by connecting the scFv-EGFR to the scDb-cMET using a (G_4_S)_3_ linker. All proteins were produced in transiently transfected FreeStyle-293F cells using polyethylenimine (PEI; branched, ∼25 kDa, Sigma-Aldrich, Burlington, MA, USA, 408727). Supernatants were harvested when cell viability dropped below 60%. Supernatants were then dialyzed against PBS overnight with a 6- to 8-kDa molecular weight cutoff and purified by IMAC followed by concentration on a VivaSpin concentrator column (MWCO 10 kDa, Sartorius, Göttingen, Germany, VS0101) for the scDb-scFv.

### Cell culture

Human cell lines A549 (lung adenocarcinoma), SK-Mel-23 (melanoma, kindly provided by Prof. Dr. Stefan Eichmüller, DKFZ, Heidelberg, Germany), SW480 (colon adenocarcinoma), H1650 (lung adenocarcinoma, kindly provided by Prof. Dr. Holger Sültmann, DKFZ, Heidelberg, Germany), and SKOV3 (ovarian serous cystadenocarcinoma) were cultivated using standard procedures. Cell lines were authenticated by SNP profiling (Multiplexion GmbH, Heidelberg, Germany).

### Generation of recombinant adenoviruses

All viruses are E3-deleted conditionally replication-competent (oncolytic) adenoviruses. The viruses have a deletion of 24 bp in the E1A gene responsible for tumor selectivity.^82^ Ad5/3 viruses have the fiber knob domain exchanged with the knob domain of Ad serotype 3 for better transduction.^78^ In Ad5/3-tE, Ad5/3-tEr, Ad5/3-DM, Ad5/3-DMr, Ad5/3-DMFE, Ad5/3-DMFEr, and Ad5/3-DMTtE, the TCE genes were inserted with an upstream CMV promoter and downstream polyadenylation sequence between the Ad E4 genes and the RITR. In Ad5/3-S-tE, the taFv-EGFR was inserted with an upstream splice acceptor sequence of the Ad serotype 40 long fiber gene downstream of the fiber gene. To enable detection of both the taFv-EGFR and the scDb-cMET in single samples, the His-tags were modified to an HA-His double tag for the taFv-EGFR and a Myc-His double tag for the scDb-cMET. Virus genomes were cloned using homologous recombination in bacteria as described.^89^^,^^92^ Virus particles were rescued in A549 cells, amplified in these cells, purified, and titrated as described before.^89^ Genome identity and integrity was determined by PCR, restriction digests, and sequencing. For ratios of virus particles (determined by OD^260^ measurement) to infectious virus particles (determined by TCID_50_ assay) of virus preparations see Figure S2.

### Biochemical characterization of T cell engagers

Purified proteins were analyzed by SDS-PAGE under reducing conditions and stained with Coomassie Brilliant Blue G-250. Purity and integrity were determined via size-exclusion chromatography using an HPLC machine (VANQUISH; Thermo Fisher Scientific) with the column Superdex 200 Increase (8.6 μm, 7.8 mm × 300 mm; GE HealthCare, Chicago, IL, USA).

### Binding analysis of purified antibodies

1 × 10^5^ target cells were incubated with serial dilutions of recombinant proteins for 45 min at 4°C. Bound protein was detected using an anti-His PE-conjugated mAb (Miltenyi Biotec, Bergisch Gladbach, Germany). Incubation and washing steps were conducted in PBS, 2% fetal calf serum (FCS), and 2 mM EDTA. Fluorescence was measured via flow cytometry, and data were analyzed using FlowJo (FlowJo LLC, Ashland, OR, USA). Relative MFI (rel. MFI) was calculated as follows: rel. MFI = ((MFI_sample_ – (MFI_detection_ – MFI_cells_))/MFI_cells_).

### Cytotoxicity assays with recombinant proteins

Target cells (2 × 10^4^ cells/well, 1.5 × 10^4^ for SK-Mel-23) were incubated 1 day post seeding with recombinant proteins for 15 min at room temperature before addition of PBMCs (E:T ratio 10:1). PBMCs were isolated from buffy coats of healthy donors via density gradient centrifugation. After 3 days, supernatants were discarded and cells were stained with 0.05% crystal violet in 20% methanol. Staining was dissolved in 10% acetic acid (50 μL/well), diluted 1:10, and absorbance was measured at 590 nm using Multiskan SkyHigh microplate spectrophotometer (Thermo Fisher Scientific).

### Virus-mediated cytotoxicity assays

2 × 10^4^ tumor cells per well in 48-well plates were infected in RPMI/2% FCS with adenoviruses from 0.0016 to 5 TCID_50_/cell (MOI) in 5-fold serial dilutions or were mock infected. Cells were incubated until cytopathic effect was observed for the lowest MOI for one virus. Supernatants were then discarded and cells were stained with crystal violet.

### Quantification of virally produced antibodies

2 × 10^4^ target cells per well in 96-well plates were infected with MOI 5 (SW480 and SKOV3) and MOI 25 (SK-Mel-23). Seven days post infection, supernatants were taken for analysis via flow cytometry. 1 × 10^5^ cells were incubated with supernatants in a dilution series for 45 min at 4°C. Bound protein was detected using an anti-His PE-conjugated mAb (Miltenyi Biotec), or in case of expression of two antibodies, an anti-HA PE-conjugated mAb (Miltenyi Biotec) for taFv-EGFR and an anti-Myc AF647-conjugated mAb (BioLegend, San Diego, CA, USA) for scDb-cMET were used. Incubation and washing steps were conducted in PBS, 2% FCS, and 2 mM EDTA. Fluorescence was measured via flow cytometry, and data were analyzed using FlowJo. Relative MFIs (calculated as described above) were used to calculate TCE concentrations based on standard curves generated with titrations of purified antibodies with known concentrations.

### Early activation of T cells

2 × 10^4^ target cells per well in 96-well plates were seeded and infected with oAds on the following day with MOI 1 for SW480 and SKOV3 and MOI 5 for SK-Mel-23. One day post infection, PBMCs were added in an E:T ratio of 3:1 for SW480 and SKOV3 and 6:1 for SK-Mel-23. Early activation was determined by CD69 expression. To that end, PBMCs were harvested after 24 h and CD69-expressing CD4^+^ and CD8^+^ T cells were identified via flow cytometry. Effector cells were stained for CD3, CD4, CD8, and CD69 (anti-CD3 AF700 [BioLegend], anti-CD4 PE [BioLegend], anti-CD8 APC [BD Pharmingen, San Diego, CA, USA], and anti-CD69 AF488 [BioLegend]). Data were analyzed using FlowJo.

### IL-2/IFNγ release assay

2 × 10^4^ target cells per well in 96-well plates were seeded and infected with oAds on the following day with MOI 1 for SW480 and SKOV3 and MOI 5 for SK-Mel-23. One day post infection, PBMCs were added in an E:T ratio of 3:1 for SW480 and SKOV3 and 6:1 for SK-Mel-23. After 24 h (IL-2) or 48 h (IFNγ), cell-free supernatants of the co-cultures were harvested and IL-2/IFNγ concentrations were determined by sandwich ELISA using ELISA MAX Sets (ELISA MAX Deluxe Set Human IL-2; ELISA MAX Standard Set Human IFN-γ; BioLegend) following the manufacturer’s instructions.

### Cytotoxicity in co-cultures of oAd-infected tumor cells and PBMCs

Previously seeded 2 × 10^4^ tumor cells per well in 96-well plates were infected the following day with oAds at stated MOIs selected based on pilot experiments, exhibiting best suitability for quantification of combined oncolytic and TCE-mediated effect. PBMCs were added in an E:T ratio of 10:1 for SK-Mel-23 and 3:1 for SKOV3, A549, and H1650 one day post infection. For SW480, PBMCs were added in an E:T ratio of 10:1 three days post infection. Six days post infection, supernatants were discarded and cells were stained with crystal violet. Crystal violet was dissolved and quantified as described above.

### Statistics

All data are presented as mean ± SD. Significances were calculated by GraphPad Prism 10 (Dotmatics, Boston, MA, USA), and results were compared by *t* test or one-way ANOVA, as indicated.

## Data availability

All data utilized in support of the findings of this study may be made available from the corresponding author upon reasonable request.

## Acknowledgments

We thank Celine Bauer and Joshua Hesse for technical help and discussions and Claudia Tessmer, Matthias Utz, and Fabian Zimmermann of the DKFZ Antibody Core Facility for their help in antibody production. We are grateful to Prof. Dr. Holger Sültmann for providing the H1650 cell line and Prof. Dr. Stefan Eichmüller for providing the SK-Mel-23 cell line. We thank Sabine Münkel for technical assistance performing HPLC analyses and Dr. Jonny Hertzog for fruitful discussions and advice during revision of the manuscript.

This work was funded by the German Academic Scholarship Foundation (10.13039/501100004350Studienstiftung des Deutschen Volkes, PhD fellowship to M.A.B.) and the Heidelberg Graduate Academy (completion grant to M.A.B.).

## Author contributions

Conceptualization, M.A.B., D.M.N., O.S., R.E.K., and G.U.; methodology, M.A.B., D.M.N., O.S., I.H., and R.E.K.; investigation, M.A.B., O.S., J.G., and S.S.; formal analysis, M.A.B., D.M.N., O.S., and R.E.K.; visualization, M.A.B.; supervision, D.M.N. and R.E.K.; validation, M.A.B., D.M.N., O.S., and R.E.K.; writing – original draft, M.A.B. and D.M.N.; writing – review & editing, M.A.B., D.M.N., G.U., O.S., and R.E.K.

## Declaration of interests

R.E.K. is a consultant for Roche, Immatics, BioCopy, BioNTech, SunRock, and Oncomatryx. O.S. is a consultant for SunRock. G.U. is a founder, CMO/CSO, and stakeholder of CanVirex.

## Supplemental information

Document S1. Figures S1–S5

Document S2. Article plus supplemental information
