# Supplementary material for: Oncolytic adenoviruses encoding bispecific T cell engagers or a novel trispecific T cell engager for dual-targeting of c-MET and EGFR
Source: Mol Ther Oncol. 2025 Dec 9;34(1):201106. doi: 10.1016/j.omton.2025.201106 (PMC12804148; doi:10.1016/j.omton.2025.201106)
Supplement: Document S1. Figures S1–S5 [file mmc1.pdf]

**OMTON, Volume *34***

## Supplemental information

# Oncolytic adenoviruses encoding bispecific T cell engagers or a novel trispecific T cell engager for dual-targeting of c-MET and EGFR

**Martin A. Boos, Oliver Seifert, Stefanie Sawall, Jessica Genz, Annika Huber, Ilse Hofmann, Roland E. Kontermann, Guy Ungerechts, and Dirk M. Nettelbeck**

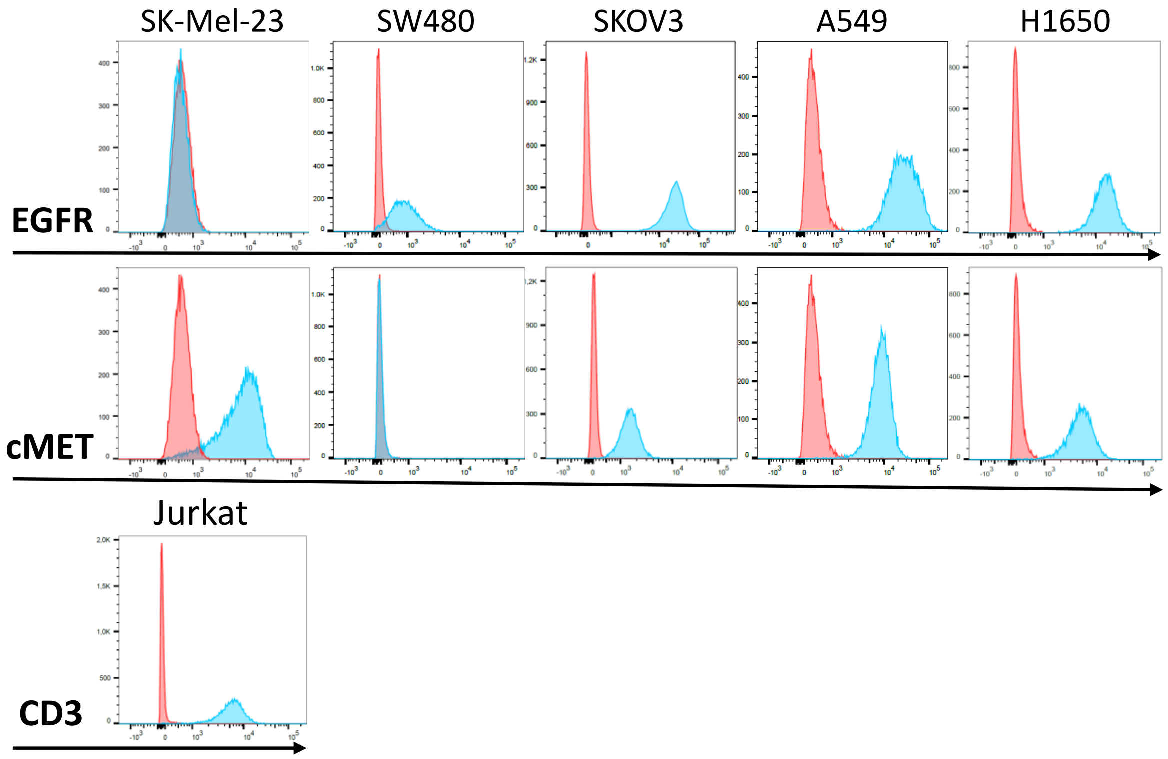


**Figure S1 EGFR- and cMET-expression on target cells and CD3-expression on Jurkat cells.** Receptor expression analyzed via flow cytometry using the same concentrations of specific mAbs and secondary APC-anti-IgG-Fc antibody.

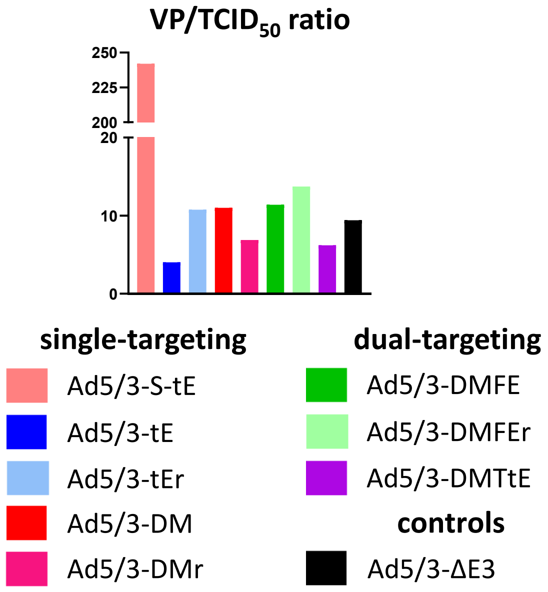


**Figure S2 Ratios of physical virus particles (VP) to infectious virus titer**. Infectious titers measured via tissue culture infectious dose 50 ($TCID_{50}$) assay.

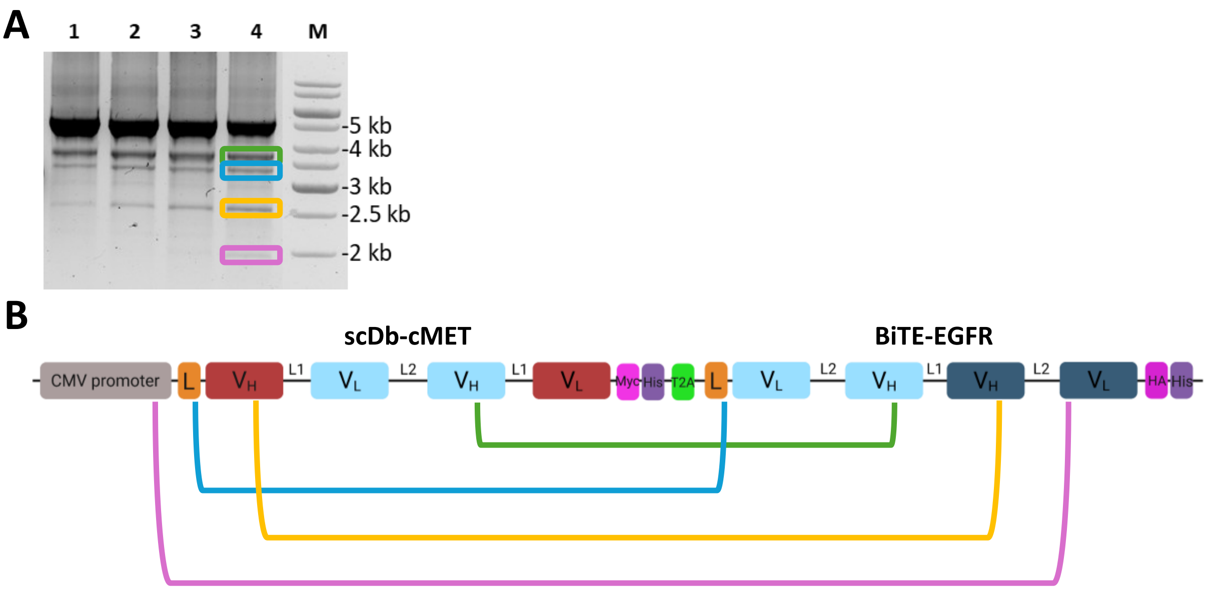


**C**

**Deletion 1:**

Amino acid sequence:

$V_H$CD3 of scDb …RSEDTAVYYC…
$V_H$CD3 of BiTE …RSEDTAVYYC…

DNA sequence:

$V_H$CD3 of scDb …CGGAGCGAGGACACCGCCGTGTACTATTGC…
$V_H$CD3 of BiTE …AGAAGCGAGGACACCGCCGTGTACTACTGT…

**Deletion 2:**

Amino acid sequence:

Igκ leader of scDb …METDTLLLWV…
Igκ leader of BiTE …METDTLLLWV…

DNA sequence:

Igκ leader of scDb …ATGGAGACAGACACACTCCTGCTATGGGTA…
Igκ leader of BiTE …ATGGAGACAGACACACTCCTGCTATGGGTA…

**Deletion 3:**

Amino acid sequence:

$V_H$cMET …LQMNSLRAED…
$V_H$EGFR …LQMNSLRAED…

DNA sequence:

$V_H$cMET …CTGCAGATGAACAGCCTGCGGGCCGAGGAC…
$V_H$EGFR …CTGCAGATGAACAGCCTGCGTGCGGAAGAT…

**Deletion 4:**

DNA sequence:

CMV-promoter …TGGGCGGTAGGCGTG-TAC…
$V_L$EGFR …TGGGCGAT---CGTGTTAC…

**Figure S3 Deletion of sequence in Ad5/3-DMTtE (Ad5/3-CMV-scDb-cMET-T2A-taFv-EGFR).** A) Gel electrophoresis of PCR amplicons in 1 % agarose gel. 1 - purified virus; 2 - passage one, 4 dpi; 3 - passage two, 9 dpi (cumulative); 4 - passage 3, 14 dpi (cumulative). Colored rectangles mark sequenced bands, corresponding to colored parentheses in B. B) Annotated insert area of oAd genome. Parentheses mark deleted areas as confirmed via Sanger sequencing. C) Amino acid and DNA sequences of the beginning of the homologous sequences for each deletion. The text color of deletions 1-4 corresponds to A and B. Non-homologous nucleotides are marked red.

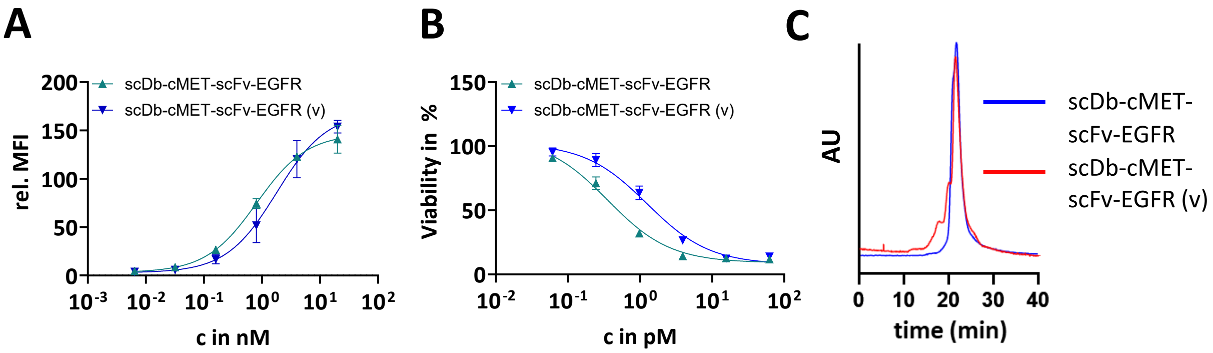


**Figure S4 Characterization of virally produced scDb-cMET-scFv-EGFR.** A) Binding properties of scDb-cMET-scFv-EGFR purified from supernatant of transfected HEK293 cells compared to scDb-cMET-scFv-EGFR purified from supernatant of oAd-infected A549 cells (v). Binding was analyzed using SKOV3 cells. Bound protein was detected using PE-anti-His mAb via flow cytometry. Relative MFI shown as mean ±SD, (n=2). B) Induction of T-cell cytotoxicity of virally (v) and non-virally produced scDb-cMET-scFv-EGFR on SKOV3 cells. Cells were incubated with a serial dilution of scDb-cMET-scFv-EGFR. PBMCs were added in an effector:target cell ratio of 10:1. Cell viability was measured 3 days after PBMC addition using crystal violet staining and subsequent quantification. Three independent experiments with different donors were conducted, data shown as mean ± SD, (n=3). C) Size-exclusion chromatography by HPLC using Superdex™ 200 Increase column.

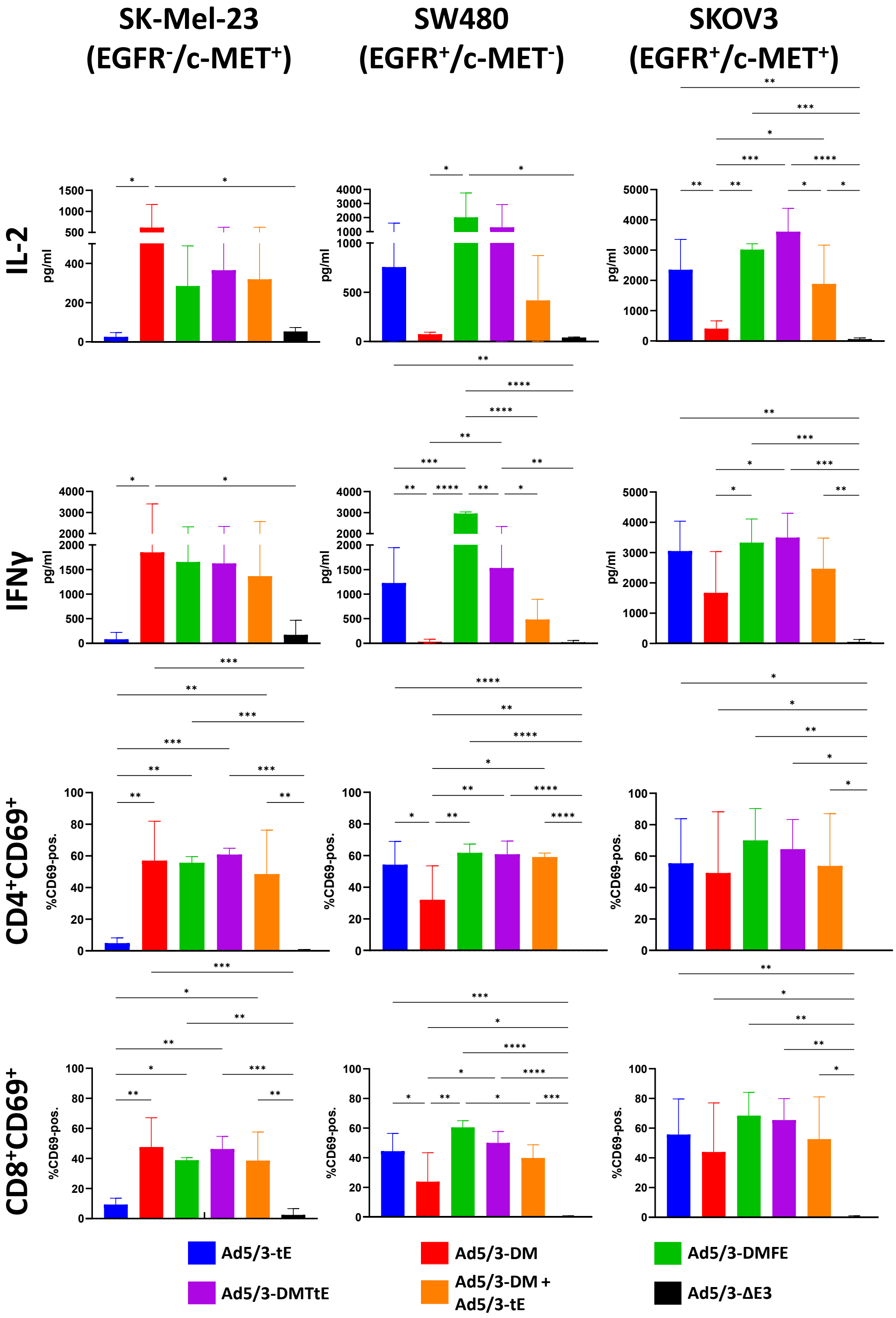


**Figure S5 T-cell activation by antibody-encoding oAds in co-cultures of infected tumor cells and PBMCs.** Tumor cells were infected with MOI 5 (SK-Mel-23) or MOI 1 (SW480, SKOV3). PBMCs were added 1 day post infection, IL-2 concentrations in the supernatant and CD69 surface expression were measured 24h post addition of PBMCs. IFNγ concentrations in supernatants were measured 48h post addition of PBMCs. Cytokine levels were measured via ELISA, CD69-expression via flow cytometry. Data gathered from 3 independent experiments with different donors, values shown as mean ± SD (n=3), *P < 0.05, **P < 0.01, ***P < 0.001, ****P < 0.0001, one-way ANOVA.
